# Hepatitis B vaccination status among health workers in Nigeria: a nationwide survey between January to June 2021

**DOI:** 10.1186/s13690-023-01142-y

**Published:** 2023-07-04

**Authors:** Amudalat Issa, Yekeen Ayodele Ayoola, Mohammed Baba Abdulkadir, Rasheed Olayinka Ibrahim, Tijani Idris Ahmad Oseni, Musa Abdullahi, Rasheedat Mobolaji Ibraheem, Aishat Funmi Lawal, Bolade Folasade Dele-ojo, Bilkis Iyabo Owolabi, Chidiebere Peter Echieh

**Affiliations:** 1 Department of Paediatrics, Children Specialist Hospital, Ilorin, Nigeria; 2 Department of Medicine, Gombe State University, Gombe, Nigeria; 3 Department of Paediatrics & Child Health, University of Ilorin Teaching Hospital, Ilorin, Nigeria; 4 Department of Family Medicine, Ambrose Alli University, Ekpoma, Nigeria; 5 Department of Paediatrics, Amadu Bello University, Zaria, Nigeria; 6 Department of Medicine, Ekiti State University Teaching Hospital, Ado-Ekiti, Nigeria; 7 Department of Paediatrics, General Hospital, Ilorin, Nigeria; 8 Department of Surgery, University of Calabar, Calabar, Nigeria

**Keywords:** Hepatitis B, Vaccination, Healthcare workers, Nigeria

## Abstract

**Background:**

Hepatitis B vaccination is the most important preventive measure against Hepatitis B viral (HBV) infection. Vaccination against HBV infection among healthcare workers is important because of their daily exposure to patients’ body fluids and the possible risk of transmission to other patients. Hence, this study assessed the risk of hepatitis B infection, vaccination status and associated factors among healthcare workers in six geopolitical zones of Nigeria.

**Methods:**

A nationwide cross-sectional study was conducted between January and June 2021 using electronic data capture techniques to enroll 857 healthcare workers (HCWs) in regular contact with patients and their samples through a multi-stage sampling method.

**Results:**

The participants’ mean (SD) age was 38.7 (8.0) years, and 453 (52.9%) were female. Each of the six geopolitical zones in Nigeria had a fair representation, with a range of 15.3 to 17.7% of the study population. The majority (83.8%) of healthcare workers in Nigeria knew that they were at an increased risk of being infected by their work. Also, 72.2% knew that if infected, there was a high risk of liver cancer in later life. Many participants (642 [74.9%]) responded that they consistently applied standard precautions, such as hand washing, gloves, and face masks while attending to patients. Three hundred and sixty (42.0%) participants were fully vaccinated. Of the 857 respondents, 248 (28.9%) did not receive any dose of the hepatitis B vaccine. Factors that were associated with not being vaccinated included age less than 25 [adjusted odds ratio (AOR) 4.796, 95% CI 1.119 to 20.547, *p* = 0.035], being a nurse (AOR 2.346, 95% CI 1.446 to 3.808, *p* = 0.010), being a health attendant (AOR 9.225, 95% CI 4.532 to 18.778, *p* = 0.010), and being a healthcare worker from the Southeast (AOR 2.152, 95% CI 1.186 to 3.904, *p* = 0.012) in Nigeria.

**Conclusion:**

This study showed a high level of awareness of the risks associated with hepatitis B infection and suboptimal uptake of the hepatitis B vaccine among healthcare workers in Nigeria.

**Table:** 

**Text box 1. Contribution to knowledge**
• Nigeria is one the low-middle income countries with endemic hepatitis B infection with healthcare workers at a high risk.
• This study shows a high level of awareness of hepatitis B viral infection and associated complications, such as hepatocellular cancer.
• The number of healthcare workers that complete vaccination for hepatits B is low.
• Main reasons for non-completion or unvaccinated included the cost of the vaccines, lack awareness of the total dose required for complete vaccination and forgetfulness.

## Background

Hepatitis B virus (HBV) is a highly infectious viral pathogen of public health significance, with the potential to cause fulminant hepatic failure [1]. It is a major risk factor for liver cirrhosis and hepatocellular cancer [1–3]. The available report indicates that approximately two billion people are infected with HBV worldwide, with the majority in Africa and Asia [4]. In addition, 240 million of the world’s population have chronic hepatitis B viral infection [4, 5].

The mode of transmission of hepatitis B virus commonly occurs through contact with body fluids such as blood, semen, and vaginal secretions. Transmission of the virus may also result from accidental inoculation of minute amounts of blood or fluid during medical, surgical, and dental procedures [6, 7]. In addition, the virus is highly transmittable and remains infectious for up to seven days on environmental surfaces, even without visible bloodstains [8]. By the nature of their work, healthcare workers are at an increased risk of HBV infection because of the day-to-day risk of exposure to patients and their body fluids [8–10]. Although standard precautions and post-exposure prophylaxis reduce the risk of HBV infection in at-risk populations, including healthcare workers, active immunization remains the most important preventive measure [1].

Despite the availability of the HBV vaccine, previous studies have reported low uptake of the hepatitis B vaccine among HCWs, especially in low- and middle-income countries [11]. Complete vaccination (at least three doses) against hepatitis B virus ranged from 36.2 to 59.5% in Nigeria, with the highest rate among doctors [12–14]. In Nigeria, policy guidelines for hepatitis B prevention advocate and recommend vaccinating all healthcare workers for the viral infection. Although HBV vaccines are expected to be available to all health care workers, their supply and availability are usually erratic. Atimes, the healthcare workers bear the cost of the vaccines. Thus, there is variability in the uptake of HBV vaccines in the country. Available studies on HBV vaccination coverage among healthcare workers (HCWs) are limited to some parts of Nigeria, and were predominantly from tertiary healthcare facilities, with a complete vaccination rates of 36.2 to 59.4% [12–14]. Considering the high prevalence in Nigeria [4] and the significant risk among HCWs, assessing the risk perception and vaccination status and associated factors among HCWs at the national level is necessary. This will provide data that may help suggest recommendations for modifying health programs and policy formulation. Therefore, this study aimed to assess the risk perception of HCWs toward hepatitis B virus infection, vaccination status and associated factors among healthcare workers in six geopolitical zones of Nigeria. We also documented the reasons for no or partial vaccination among HCWs in Nigeria.

## Methods

### Study design and site

This nationwide cross-sectional study was conducted from January to June 2021 using electronic data capture techniques (Google Form). All six geopolitical zones in Nigeria had a fairly equal representation.

### Inclusion and exclusion criteria

This study included all HCWs in regular contact with patients and their body fluid samples (doctors, nurses, health attendants, laboratory scientists, and technicians) who consented to participate. Pharmacists and administrative staff were excluded because of their reduced risk of exposure to body fluids.

### Sample size estimation

We estimated the minimum sample size of 600 using a prevalence of (48.5%) complete hepatitis B vaccination observed among HCWs in Lagos, Southwestern Nigeria [14], at a 95% confidence level and a precision of 4%; a total of 857 participants were finally recruited.

### Sampling technique

A multi-stage probability sampling method was used to select study participants as follows:

Stage I: Based on the six geopolitical zones in Nigeria, we allocated an equal minimum number of participants (100) per zone. A simple random method was used to select a state in each zone. A simple random selection was used in each state to select a senatorial district out of three.

Stage II: In the selected senatorial district, a list of secondary and tertiary health facilities was obtained from the state health ministry. Afterwards, one secondary and tertiary health facilities were selected via a simple ballot. Where there was only a secondary or tertiary health facility in the Senatorial District, this was selected to represent the state.

Stage III: Based on the population size of the HCWs in the selected hospitals, a proportionate number of study participants were allocated to the two health facilities. Each health facility’s allocation was equally distributed based on cadres (doctors, nurses, health attendants, laboratory scientists, and technicians).

### Data collection

A pre-tested questionnaire on a “Google Form” template was used to collect data via social media platforms. The data collected included sociodemographic features such as age, sex, level of education, cadre of HCWs, duration of work, and place of work. Risk perception was ascertained by whether the subjects considered themselves to be at risk of HBV infection, the frequency of exposure to body fluids, the risk of liver cancer, and the importance of HBV vaccination. Furthermore, the responses to the HBV vaccination status of each participant were ascertained. The participants self-administered the questionnaire with a trained interviewer administered in a situation of low literacy level (attendants who cannot read and write) or limited or no Internet access, especially among lower-cadre HCWs.

### Data analysis

The obtained information was transferred and analyzed using the Statistical Package for the Social Sciences software version 23 (SPSS Inc., USA). Age was summarized as means with standard deviations (normal distribution) and further grouped into three categories (less than 25, 25–45, and > 45 years) for analysis. Frequency tables and charts were used to summarize the sociodemographic characteristics and doses of hepatitis B vaccines received. Binary logistic regression was used to determine variables that independently predicted partial or no vaccination and expressed as an adjusted odds ratio (AOR) with a 95% confidence interval. For all tests, a p-value of less than 0.05 was considered significant.

### Ethical considerations

Ethical approval was obtained from the institutional review board of the states participating in the study. Informed consent was obtained from all participants and the study protocol conformed to the ethical guidelines of the 1975 Declaration of Helsinki. The Google Form was designed such that after the initial study information, the participants either picked yes or no for consent and declined, respectively. Those who picked yes will progress to the next stage of filling out the proforma, and those who picked no will have the form close automatically.

## Results

### General characteristics of the study population

This study recruited 857 HCWs across six geopolitical regions of Nigeria (six tertiary and six secondary health facilities). The mean age was 38.7 ± 8.0 years, and most participants were female (453; 52.9%). Most HCWs (81.9%) were aged between 25 and 45 years (Table 1). Almost all HCWs (93.2%) had tertiary education. Of the study participants, 36.1% were nurses and 35.2% were medical doctors. Approximately half of the HCWs (49.2%) had spent more than 10 years in practice (Table 1).

**Table 1:** General characteristics of the study population*

Variable	Frequency (n = 857)	Per cent (%)
**Age (Years)**		
Less than 25	17	2.0
25–45	702	81.9
Greater than 45	138	16.1
**Gender**		
Male	404	47.1
Female	453	52.9
**Level of education**		
Primary	18	2.1
Secondary	40	4.7
Tertiary	799	93.2
**Designation**		
Doctor	302	35.2
Nurse	309	36.1
Lab. Scientist/Technician	112	13.1
Health attendants	134	15.6
**Region**		
North Central	143	16.7
North East	152	17.7
North West	150	17.5
South South	137	16.0
South East	131	15.3
South West	144	16.8
**Number of years in practice**		
Less than 5 years	228	26.6
5 to 10 years	207	24.2
Greater than 10 years	422	49.2

Lab: Laboratory; *Healthcare workers in secondary and tertiary facilities across the six geopolitical zones in Nigeria from January to June 2021

### HBV risk perception among healthcare workers

Overall, a good perception of HBV risk was found among HCWs in Nigeria, as 82.1% knew they were at an increased risk of being infected at work. In addition, most (72.2%) knew that if infected, there is a high risk of liver cancer later in life. Only 2.9% of the HCWs did not believe in the HBV vaccine (Table 2).

**Table 2:** HBV risk perception among healthcare worker in secondary and tertiary health facilities in Nigeria from January to June 2021

Variables	n	I am at increased risk of getting a hepatitis B infection by my work		If I am infected, there is a high risk of liver cancer later in life.		I do not believe in the hepatitis B vaccine	
		Yes	%	Yes	%	Yes	%
**All HCW**	857	704	82.1	619	72.2	25	2.9
**Doctors**	302	253	83.3	264	87.4	0	0.0
**Nurses**	309	263	85.1	230	74.4	5	1.6
**Lab. Sci/Tech.**	112	103	92.093.9	72	64.3	9	8.0
**Health att.**	85	49	63.4	53	39.6	11	8.2

HCW-Healthcare workers; Lab Sci-Laboratory scientists; Lab Tech-Laboratory technologists; Health att: Health attendants

### Practices/Attitudes of healthcare workers towards HBV Prevention

More than half of the HCWs reported that they always or usually had exposure to blood products and body fluids in the hospital. The majority of participants (74.9%) said they always applied standard precautions (the minimum infection prevention practices that apply to all patient care, regardless of the suspected or confirmed infection status of the patient, in any setting where health care is delivered, for example, regular hand washing, use of protective gloves, and face masks while attending to patients depending on the level of exposure) while attending to patients and their body fluids, while 39.4% said they might not find the time to get vaccinated with the HBV vaccine, as shown in Table 3.

**Table 3:** Practices/Attitudes of healthcare workers towards HBV Prevention among healthcare worker in secondary and tertiary health facilities in Nigeria from January to June 2021

Variables	I do have exposure to blood products and body fluid in the hospital		I do apply standard precautions while attending to patients and body fluid		I may not find the time to get the hepatitis B vaccine	
n = 857	Yes	%	Yes	%	Yes	%
Always	306	35.7	642	74.9	26	3.0
Usually	215	25.1	168	19.6	121	14.1
Sometimes	312	36.4	44	5.1	338	39.4
Never	24	2.8	3	0.4	372	43.4

### HBV vaccination status among study participants

**Fig. 1:**
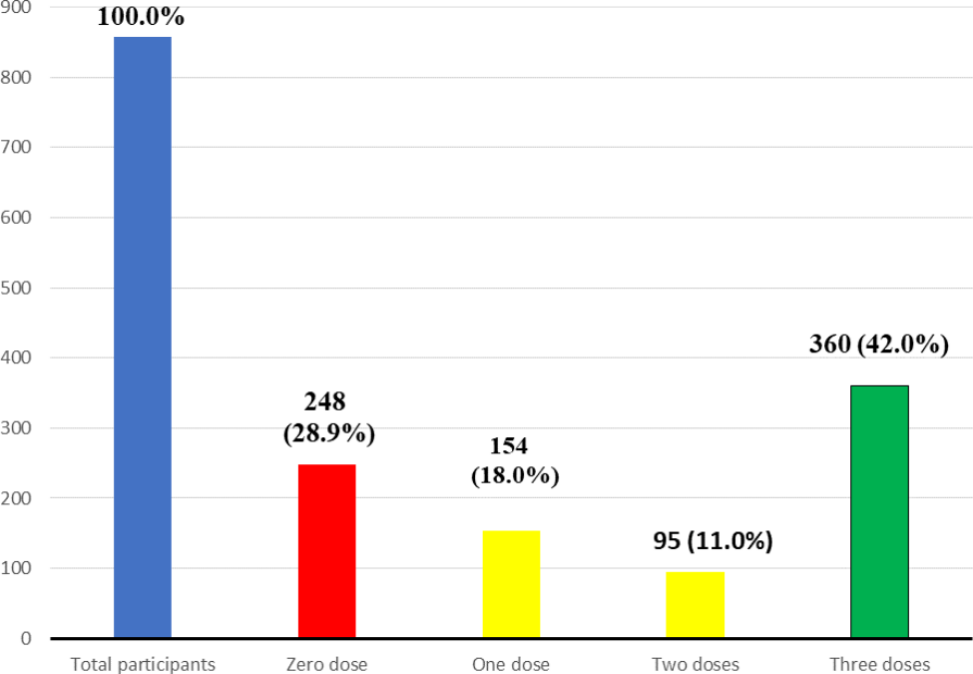
Bar chart showing the number of doses (Hepatitis B vaccine) in the participants across the secondary and tertiary health facilities in Nigeria from January-June 2021

The majority [609 (71.1%)] of the HCWs received at least one dose, with 360 (42.0%) of the received three doses (full vaccination), while 248 (28.9%) were not vaccinated, as shown in Fig. 1.

### Risk factors associated with non-vaccination among healthcare workers

Of the 857 participants, 248 (28.9%) did not receive any dose of the hepatitis B vaccine. Factors that were associated with not being vaccinated included age less than 25 with an AOR of 4.796, (95% CI 1.119 to 20.547, *p* = 0.035), being a nurse with an AOR of 2.346 (95% CI 1.446 to 3.808, *p* = 0.010), being a health attendant with an AOR of 9.225 (95% CI 4.532 to 18.778, *p* = 0.010), and being a healthcare worker from the Southeast with an AOR of 2.152, (95% CI 1.186 to 3.904, p = 0.012). However, sex, educational level, and years of practice did not have a significant relationship with the risk of non-vaccination against hepatitis B virus (Table 4).

**Table 4:** Risk factors associated with non-vaccination among healthcare workers among healthcare worker in secondary and tertiary health facilities in Nigeria from January to June 2021

Variables	Unvaccinated N = 248	OR	95%		AOR	95%		P value
**Age (Years)**								
Less than 25	10	4.167	1.086	15.990	4.796	1.119	20.547	**0.035**
25–45	190	0.800	0.525	1.218	0.790	0.486	1.286	0.343
Greater than 45	48	1						
**Sex**								
Female	137	1						
Male	111	1.036	0.748	1.434	1.478	0.984	2.218	0.060
**Level of education**								
Primary	13	7.109	1.977	24.917	1.537	0.375	6.229	0.551
Secondary	22	2.969	1.440	6.124	0.869	0.353	2.138	0.759
Tertiary	213	1						
**Designation**								
Doctor	59	1						
Nurse	89	1.625	1.082	2.442	2.346	1.446	3.808	**0.010**
Lab. Scientist/Technician	25	0.982	0.562	1.714	1.355	0.743	2.471	0.321
Health attendants	75	5.522	3.303	9.230	9.225	4.532	18.778	**< 0.010**
**Region**								
North Central	49	1.348	0.806	2.256	1.460	0.800	2.662	0.218
North East	56	1.077	0.663	1.749	0.886	0.509	1.543	0.699
North West	46	0.966	0.584	1.600	1.732	0.991	3.027	0.054
South South	4	3.192	0.565	18.050	4.098	0.646	26.000	0.135
South East	41	1.128	0.665	1.915	2.152	1.186	3.904	**0.012**
South West	52	1						
**Number of years in the practice**								
Less than 5 years	71	1						
5 to 10 years	69	0.972	0.633	1.493	1.077	0.668	1.736	0.760
Greater than 10 years	108	1.017	0.689	1.502	0.959	0.621	1.481	0.850

Lab: Laboratory. OR-Crude odds ratio; AOR-adjusted odds ratio; dependent variable-Unvaccinated participants

### Factors associated with incomplete vaccination among healthcare workers

Of the 857 participants, 249 received at least one dose of the Hepatitis B vaccine. Age, sex, educational level, healthcare worker’s designation, region of the country, and years of practice were not associated with incomplete doses of the hepatitis B vaccine (Table 5). Of the 249 participants who did not complete the course of HBV vaccination, more than half (53.0%) of the partially vaccinated participants said they had just started the series and planned to return later for the second and third doses. Other reasons for non-completion included not being aware that three doses were required (12.9% (32/249) and forgetfulness (23.3%) (58/249). A few participants, 15 (6.0%) and 6 (2.4%) were on vacation or sick leave or had side effects from previous doses.

**Table 5:** Factors associated with incomplete vaccination among healthcare workers among healthcare worker in secondary and tertiary health facilities in Nigeria from January to June 2021

Variables	Partially vaccinated N = 249	OR	95% Confidence interval		Adjusted OR	95% confidence interval		P value
**Age (Years)**								
Less than 25	4 (1.6)	2.667	0.560	12.687	2.886	0.539	15.461	0.216
25–45	215 (86.3)	1.448	0.903	2.321	0.850	0.460	1.569	0.603
Greater than 45	30 (12.0)	1						
**Gender**								
Female	114 (45.8)	1						
Male	135 (54.3)	1.514	1.094	2.095	1.360	0.821	2.252	0.232
**Level of education**								
Primary	2 (0.8)	0.954	0.158	5.755	1.427	0.151	13.459	0.756
Secondary	6 (2.4)	0.716	0.265	1.933	1.092	0.229	5.204	0.912
Tertiary	241 (96.8)	1			1			
**Designation**								
Doctor	104 (41.8)	1						
Nurse	91 (36.5)	0.943	0.652	1.364	0.862	0.498	1.491	0.595
Lab. Scientist/Technician	27 (10.8)	0.601	0.357	1.012	0.329	0.144	0.752	**0.008**
Health attendants	27 (10.8)	1.128	0.637	1.988	0.920	0.278	3.048	0.892
**Region**								
North Central	36 (14.5)	5.724	2.562	12.787	6.976	2.979	16.335	**< 0.001**
North East	13(5.2)	1.444	0.586	3.562	1.743	0.683	4.447	0.245
North West	28 (11.2)	3.398	1.507	7.660	3.959	1.696	9.242	**< 0.001**
South South	131 (52.6)	604.056	127.360	2864.984	780.223	159.14	3823.450	**< 0.001**
South East	32 (12.9)	5.088	2.259	11.461	4.989	2.168	11.479	**< 0.001**
South West	9 (3.6)	1						
**Number of years in the practice**								
Less than 5 years	160 (64.3)	1						
5 to 10 years	54 (21.7)	0.648	0.391	1.074	1.267	0.674	2.383	0.463
Greater than 10 years	35 (14.1)	1.982	1.333	2.947	1.181	0.667	2.093	0.568

Lab: Laboratory. OR-Crude odds ratio; AOR-adjusted odds ratio; dependent variable-Unvaccinated participants

### Cost as a cause of or incomplete uptake/non-uptake of hepatitis B vaccine

A large percentage (47.2% [117] of non-vaccinated participants) said that the HBV vaccine is not free in their facility, while 37.5% (93) were unsure if it is available at no cost. About a third (40.3% (100) of the non-vaccinated participants thought that the HBV vaccine was expensive.

## Discussion

Nigeria is one of the countries classified as endemic for HBV infection, with a higher risk among healthcare workers (HCWs). The country recommends hepatitis B vaccination for all adults, including HCWs, but it is not mandatory for all HCWs in Nigeria. Hence, this study assessed the risk perception, vaccination status and associated factors among HCWs from all six geopolitical regions in Nigeria.

The mean age (38.7 ± 8.0 years) of the HCWs in this study is similar to some local studies in Nigeria, which probably reflect the middle-aged group of active service in the country [13, 15, 16]. Compared with studies outside Nigeria, the mean age is higher than 29.6 years reported in Ethiopia [17]. The relatively higher mean age obtained in this study compared to the Ethiopian study may be because the HCWs recruited in this study were from secondary and tertiary health facilities compared to the Ethiopian study, which involves all categories of health facilities. The spectrum of HCWs in this study showed that most responders were nurses, followed by doctors, similar to other works on hepatitis awareness among HCWs in Nigeria [13, 15–17]. This finding probably reflects the higher representation of nurses among HCWs in most health institutions in Nigeria.

This study demonstrates a high level of awareness about the risk and complications of hepatitis B infection among HCWs in Nigeria, consistent with earlier reports [18, 19]. The level of awareness is probably due to the increasing awareness of HBV with the yearly ‘World hepatitis day’ celebrated in most health facilities in Nigeria. The increasing number of cases and complications of Hepatitis B viral infection among HCWs and the general population may also have increased awareness of the risk associated with the infection. It is worth noting that the level of awareness of HBV infection in this study was slightly lower than the 96.0% reported by Adekanle *et al*. [13] and 91.0% reported by Abiola *et al*. [14] in tertiary health facilities in Nigeria, which may reflect differences in the study population. This study recruited all categories of HCWs in contact with patients or their body fluids compared with the study by Adekanle et al. [13], which was limited to only healthcare professionals (only clinicians who are in contact with diagnosis, treatment, and care of patients), while the Abiola et al. [14] study was carried out among doctors and nurses in Lagos Nigeria.

This study also showed that more than half of the participants had some degree of exposure to blood and body fluids. This finding is similar to observations in southeastern Nigeria, where 68% had occupational exposure at their workplace [20]. Despite the high level of exposure among HCWs, only three-quarters applies standard precautions while handling patients. These findings call for sustained health education and awareness among HCWs, including standard precautions in healthcare settings.

The level of HBV vaccination (at least one dose) in this study was similar to that in Southern Nigeria [14, 21]. However, the HBV vaccination rate in this study was far higher than the 14.2% and 6.6% reported by Omotowo et al. [22] and Orji et al. [16], respectively, in both Nigerian studies. The differences could be because the latter studies included more administrative and support staff, such as securities, who rarely have contact with patients and their body fluids, and hence pay less attention to vaccination.

The percentage of fully vaccinated HCWs (42.0%) in this study fell within the range of 13.4–62.1% reported by Auta et al. [11] in a systematic review and meta-analysis of HBV studies in African countries. This is lower than the rates of 62.5% and 59.4% reported by Abiola et al. [14].and Samuel et al. [21]., respectively, in Southern Nigeria. This study’s observed level of fully vaccinated HCWs is similar to that of a study in China. In contrast, the fully vaccinated HCWs in this study were lower than the 97.0% reported by Guthmann et al. [23] in France, probably because of well-planned and organized vaccination programs in developed countries, which include improved accessibility to the vaccine at no cost and adequate storage facilities at all levels. It is worth noting that the Nigerian government only funds the childhood hepatitis B vaccination program, which means vaccination among HCWs will bear some costs. Cost may be a limiting factor for vaccination has observed in the reasons cited for those who did not complete their vaccination. These findings call for the need to review the country’s policy that ensures free and improved and access to hepatitis B vaccine among high-risk groups, including HCWs.

This study also shows that the complete vaccination rate varies across Nigeria’s six geopolitical regions, with the highest rate in the Southwest and Northwest and the least full vaccination rate in the South-South. This observation is probably a reflection of variations in vaccination uptake in Nigeria, which tend to vary from one part of the country to another. This calls for an urgent need to strengthen awareness campaigns in areas such as the south–south and southeast of the country with fewer fully vaccinated HCWs.

This study explored the reasons for partial HBV vaccination, similar to reports by Auta et al. [11]. on a systematic review and meta-analysis on HBV in Africa. Being a laboratory techinician/scientist was a factor identified in this study that was asscoaited with incomplete vaccination. This finding may be due to the fact that this set of HCWs have a lesser contact time with patients compared with core clinical staff and runs the bench work with improved standard precautions. Similarly, the most common reasons for not vaccination were the cost and possible side effects. Another reason for non-completion is forgetfulness. An automated reminder system that leverages mobile phone use to improve childhood vaccination rates should be explored among HCWs. This will facilitate easy remembrance and improve the number of HCWs that are vaccinated.

Although our study involved all geopolitical zones of Nigeria with at least secondary and tertiary health facilities in each selected state, there are some limitations. We did not screen the participants for HBV immunoglobulin levels to correspond with their vaccination status. Instead, we relied on healthcare workers’ recall of the vaccination history regarding the HBV vaccine, which may not be 100% accurate. Also, we did not include primary healthcare facilities in our study; therefore, the findings from this study may not be extrapolated to them. In addition, although hepatitis B viral infections included liver cirrhosis, decompensated liver disease, and hepatocellular cancer, we only asked about hepatocellular cancer while evaluating awareness of the chronic complications of hepatitis B viral infection. The participants’ previous exposure to hepatitis B infection was based on a recall response to a Likert scale (always, usually, sometimes, and never) with potential bias recalls.

In conclusion, this study shows the a high level of awareness of complications associated with hepatitis B infection but but a low vaccination rate among healthcare workers, who should have a better understanding/health-seeking behavior. This suggests the need for a review of the national policy guideline on HBV vaccination in Nigeria. In addition, there is a need to encourage all HCWs to receive the HBV vaccine without hindrance or barriers, especially if the country will meets the global target of making HBV infection not a public health concern by 2030. In addition, a regular supply of free vaccines and incorporation of a reminder system may help reduce out-of-pocket spending and forgetfulness, thereby improving the number of fully vaccinated HCWs.

## Data Availability

All data will be made available upon reasonable request from the corresponding author, Dr. Issa Amudalat, Department of Pediatrics, Children Specialist Hospital, Ilorin, Nigeria.

## Abbreviations

HBV: Hepatitis B virus
HCWs: Healthcare workers
Lab: Laboratory
